# Increased risk of sudden sensorineural hearing loss in patients with hepatitis virus infection

**DOI:** 10.1371/journal.pone.0175266

**Published:** 2017-04-06

**Authors:** Hsin-Chien Chen, Chi-Hsiang Chung, Chih-Hung Wang, Jung-Chun Lin, Wei-Kuo Chang, Fu-Huang Lin, Chang-Huei Tsao, Yung-Fu Wu, Wu-Chien Chien

**Affiliations:** 1 Department of Otolaryngology-Head and Neck surgery, Tri-Service General Hospital, National Defense Medical Center, Taipei, Taiwan; 2 Department of Medical Research, Tri-Service General Hospital, National Defense Medical Center, Taipei, Taiwan; 3 School of Public Health, National Defense Medical Center, Taipei, Taiwan; 4 Division of Gastroenterology, Department of Internal Medicine, Tri-Service General Hospital, National Defense Medical Center, Taipei, Taiwan; Centers for Disease Control and Prevention, UNITED STATES

## Abstract

The etiology of sudden sensorineural hearing loss (SSNHL) remains unclear. Possible causes of SSNHL include vascular diseases, viral infection, and autoimmune disorders. Therefore, we investigated whether hepatitis virus infection is correlated with the risk of SSNHL. Using data from the Taiwan Longitudinal Health Insurance Database, we conducted a retrospective matched-cohort study to compare patients diagnosed with hepatitis B or C virus (HBV/HCV) infections from January 1, 2000, to December 31, 2010, (N = 170,942) with frequency-matched controls (N = 512,826) at a ratio of 1:3 by sex, age, and index year. We followed each patient until the end of 2010 and evaluated the incidence of SSNHL. At the end of the follow-up period, 647 (0.38%, 647/170,942) patients developed SSNHL in the HBV/HCV group compared with 978 (0.19%, 978/512,826) in the control groups, with a statistical significance of P < 0.001 (using the log-rank test). The incidence rate ratio of SSNHL was 5.743-fold higher in the HBV/HCV group than in the control group (283.17 vs. 49.31 per 100,000 person-years, P < 0.001). The risk of SSNHL increased with HBV/HCV infection, and an adjusted hazard ratio of 5.103 (95% CI, 4.585–5.678) was determined using Cox proportional hazards regression. This study contributes to the awareness of the increased risk of SSNHL in HBV/HCV-infected populations. Our findings suggest that an underlying viral infection contributes to the development of SSNHL.

## Introduction

Sudden sensorineural hearing loss (SSNHL) is often an unexpected and traumatic experience for patients. SSNHL is defined as a sudden loss of more than 30 dB of hearing acuity in three contiguous frequencies within 72 h [1]. SSNHL is usually classified as idiopathic because the causative factor is not identified in most cases [2]. The potential causes of SSNHL include vascular disorders, viral infection, autoimmune disorders, neurological disorders, neoplasms, and ototoxic drugs [3].

Several studies have discussed the presence of viral infection in patients with SSNHL [4–6], and the types of viruses implicated in such cases include mumps, cytomegalovirus, measles, rubella, varicella zoster virus, herpes simplex virus, enteroviruses, and human immunodeficiency virus [5–7]. Hepatitis virus infection has been correlated with SSNHL in only a small number of case reports [8–11]. Hepatitis B virus (HBV) and hepatitis C virus (HCV) infections have emerged as a global health problem, and as many as 350 million and 123 million individuals have been infected with HBV and HCV, respectively, worldwide [12, 13]. In highly endemic areas such as Taiwan, the prevalence of chronic HBV infection is estimated to be between 15% and 20% [14, 15], whereas the prevalence of HCV is estimated at 4%–10% in the general population [16, 17]. Chronic HBV or HCV infections have been proven to increase the risk of hepatocellular carcinoma [18]. We are interested in investigating whether HBV or HCV infections have an impact on the incidence of SSNHL.

To assess the association between HBV or HCV infection and the subsequent development of SSNHL, we conducted a nationwide population-based cohort study by analyzing data from a nationwide medical database, the National Health Insurance Research Database.

## Materials and methods

### Data sources

In this study, we used data from the National Health Insurance Research Database (NHIRD) to investigate the association between hepatitis virus infections, including HBV and HCV infection, and SSNHL over a 10-year period (2000–2010), from the outpatient and hospitalization Longitudinal Health Insurance Database in Taiwan.

### Study design and sampled participants

#### Study design

This study was a retrospective matched-cohort design.

#### Sample

**Fig 1:**
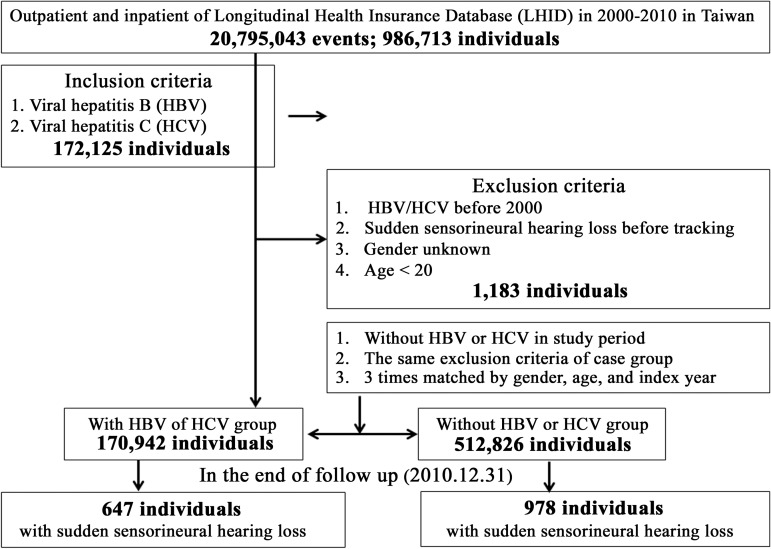
Flowchart of study sample selection from the National Health Insurance Research Database in Taiwan.

Patients with HBV or HCV infections were selected from 1 January 2000 to 31 December 2010, according to the International Classification of Diseases, Version 9, Clinical Modification (ICD-9-CM) codes 070.20, 070.22, 070.30, 070.32, and V02.61 for HBV and 070.41, 070.44, 070.51, 070.54, 070.70, 070.71, and V02.62 for HCV. Patients diagnosed with SSNHL (ICD-9-CM code: 388.2) before 2000 or before the first visit for viral hepatitis were excluded. In addition, all patients aged <20 years were also excluded. A total of 683,768 participants were enrolled in this study; among these, 170,942 had HBV or HCV infection and 512,826 were controls without HBV or HCV infection who were matched by age, sex, and index year (Fig 1).

### Covariates

The covariates included sex and age group (20–29, 30–39, 40–49, 50–59, 60–69, ≥70 years).

### Comorbidity

Baseline comorbidities included diabetes mellitus (DM; ICD-9-CM code: 250); hypertension (ICD-9-CM codes: 401–405); depression (ICD-9-CM codes: 296.2, 296.3, 296.82, 300.4, and 311); stroke (ICD-9-CM codes: 430–438); chronic kidney disease (CKD; ICD-9-CM code: 585); osteoporosis (ICD-9-CM codes: 733.00–733.09); nephritis, nephrotic syndrome, and nephrosis (ICD-9-CM codes: 580–589); hyperlipidemia (ICD-9-CM code: 272); and systemic lupus erythematosus (SLE; ICD-9-CM code: 710.0).

### Data analysis

All statistical analyses were performed using IBM SPSS for Windows, Version 22.0 (IBM Corp., Armonk, NY, USA). χ^2^ and t tests were used to evaluate the distributions of categorical and continuous variables, respectively. Multivariate Cox proportional hazards regression analysis was used to determine the risk of SSNHL, and the results are presented as hazard ratio (HR) with 95% confidence interval (CI). The difference in the risk of SSNHL between the study (with HBV/HCV) and control (without HBV/HCV) groups was estimated using the Kaplan–Meier method with the log-rank test. A two-tailed P value of <0.05 was considered to indicate statistical significance.

### Ethics statement

This study was conducted in accordance with the Code of Ethics of the World Medical Association (Declaration of Helsinki). The NHIRD encrypts personal patient information to maintain privacy and provides researchers with anonymous identification numbers associated with relevant claim information. Patient consent is not required for accessing the NHIRD. The Institutional Review Board of Tri-Service General Hospital approved this study (TSGHIRB No. 2-104-05-126). The committee waived the need for written informed consent.

## Results

### Characteristics of the prevalence of SSNHL, covariates, and comorbidities at the end of follow-up for patients with HBV/HCV infection compared with those without HBV/HCV infection

According to the assessed data from January 1, 2000, to December 31, 2010, 170,942 patients with HBV/HCV infection fulfilled the eligibility criteria and 512,826 matched individuals were selected as controls (Fig 1). In our matched-cohort study, no significant differences were observed between the HBV/HCV and control groups in sex and age distribution at baseline. At the end of follow-up (Table 1), 647 (0.38%, 647/170,942) patients developed SSNHL in the HBV/HCV group compared with 978 (0.19%, 978/512,826) in the control group; this difference was statistically significant (P < 0.001). Additionally, in the HVB/HCV group, a higher rate of the participants was observed among those in the 20–59-year age group (58.39% vs. 51.37%) and those with some comorbidities (depression, 1.09% vs. 0.81%; SLE, 0.18% vs. 0.13%) compared with the control group.

**Table 1:** Characteristics of the study participants at the end of follow-up.

HBV / HCV	Total		With		Without		P
Variables	n	%	n	%	n	%	
**Total**	683,768		170,942	25.00	512,826	75.00	
**SSNHL**							<0.001
Without	689,143	99.76	170,295	99.62	518,848	99.81	
With	1,625	0.24	647	0.38	978	0.19	
**Gender**							0.999
Male	440,600	64.44	110,150	64.44	330,450	64.44	
Female	243,168	35.56	60,792	35.56	182,376	35.56	
**Age group (years)**							0.036
20–29	37,142	5.42	10,218	6.96	25,124	4.90	
30–39	76,708	11.19	20,367	11.79	56,341	10.99	
40–49	112,488	16.41	30,710	17.78	81,778	15.95	
50–59	137,940	20.12	37,765	21.86	100,175	19.53	
60–69	132,244	19.29	33,033	19.12	99,211	19.35	
≧70	189,046	27.58	38,849	22.49	150,197	29.29	
**Diabetes mellitus (DM)**							<0.001
Without	579,370	84.73	145,758	85.27	433,612	84.55	
With	104,398	15.27	25,184	14.73	79,214	15.45	
**Hypertension**							<0.001
Without	568,594	83.16	144,676	84.63	423,918	82.66	
With	115,174	16.84	26,266	15.37	88,908	17.34	
**Depression**							<0.001
Without	677,732	99.12	169,073	98.91	508,659	99.19	
With	6,036	0.88	1,869	1.09	4,167	0.81	
**Stroke**							<0.001
Without	635,547	93.00	164,379	96.36	471,168	91.88	
With	47,861	7.00	6,203	3.64	41,658	8.12	
**Chronic Kidney Disease (CKD)**							<0.001
Without	663,782	97.08	166,862	97.61	496,920	96.90	
With	19,986	2.92	4,080	2.39	15,906	3.10	
**Osteoporosis**							<0.001
Without	683,543	99.57	170,492	99.74	513,051	99.52	
With	2,925	0.43	450	0.26	2,475	0.48	
**Nephritis, nephrotic syndrome, and nephrosis**							<0.001
Without	638,130	93.33	160,600	93.95	477,530	93.12	
With	45,638	6.67	10,342	6.05	35,296	6.88	
**Hyperlipidaemia**							<0.001
Without	666,821	97.52	167,716	98.11	499,105	97.32	
With	16,947	2.48	3,226	1.89	13,721	2.68	
**Systemic lupus erythematosus (SLE)**							<0.001
Without	682,811	99.86	170,632	99.82	512,179	99.87	
With	957	0.14	310	0.18	647	0.13	

P-value (categorical variable: chi-square/Fisher exact test). DM: ICD-9-CM code, 250; Hypertension: ICD-9-CM codes, 401–405; Depression: ICD-9-CM codes, 296.2–296.3, 296.82, 300.4, and 311; Stroke: ICD-9-CM codes, 430–438; CKD: ICD-9-CM code, 585;Osteoporosis: ICD-9-CM code, 733.0x; Nephritis, nephrotic syndrome, and nephrosis: ICD-9-CM codes, 580–589; Hyperlipidemia: ICD-9-CM code, 272; SLE: ICD-9-CM code, 710.0. HBV, hepatitis B virus; HCV, hepatitis C virus.

### Kaplan–Meier model for cumulative risk of SSNHL in HBV/HCV infection

**Fig 2:**
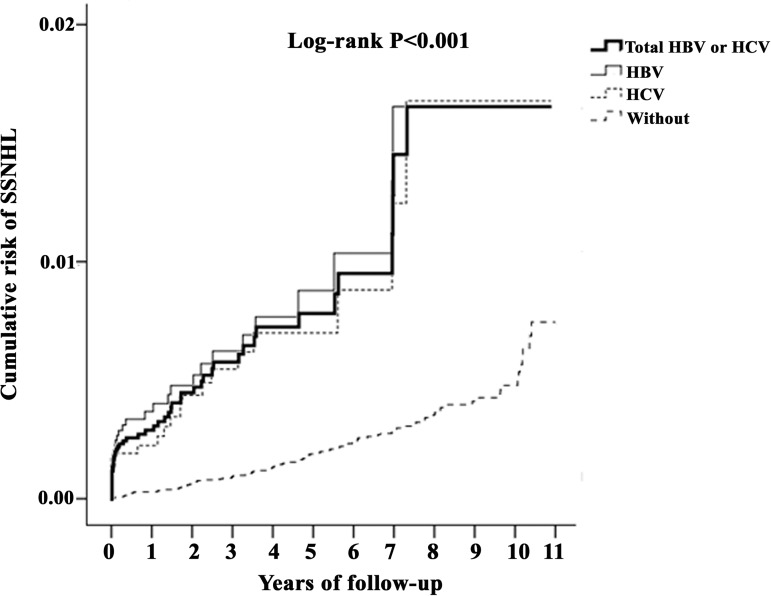
Kaplan–Meier curves for the cumulative risk of SSNHL among patients aged ≥20 years with HBV/HCV infection (stratified by HBV or HCV alone, using the log-rank test).

The cumulative incidence curve for SSNHL in the total HBV/HCV infection cohort was significantly higher than that for the comparison cohort, after adjustment for age, sex, and comorbidities (Fig 2, log-rank test; P < 0.001). In the patients with HBV/HCV infections, the risk of SSNHL increased progressively with the duration of follow-up, rather than with being limited to the immediate days after a diagnosis of HBV/HCV infection. In the subgroups of HBV and HCV infection, the same trend was independently revealed (Fig 2).

### HRs and incidence of SSNHL stratified by sex, age group, and comorbidities, by using Cox regression for patients with HBV/HCV infection compared with those without HBV/HCV infection

Cox proportional hazards regressions analysis revealed an increased risk of SSNHL, with an adjusted HR of 5.103 (95% CI, 4.585–5.678), in patients with HBV/HCV infection, after adjustment for sex, age, and comorbidities (Table 2). Adjusted HRs of 4.825 (95% CI, 4.216–5.521) and 5.582 (95% CI, 4.686–6.651) for developing SSNHL were observed in male and female patients with HBV/HCV infection, respectively, compared with the control group (P < 0.001). Among all age groups, a higher adjusted HR for SSNHL was observed in the HBV/HCV group than in the control group. Regardless of the presence of the comorbidities of DM; hypertension; depression; stroke; CKD; osteoporosis; nephritis, nephrotic syndrome, and nephrosis; and hyperlipidemia, a higher adjusted HR (ranging from 2.356 to 15.114) was observed in patients with HBV/HCV infection than in the controls (P < 0.001).

**Table 2:** Factors for sudden sensorineural hearing loss at the end of follow-up, stratified by variables assessed through Cox regression analysis.

HBV / HCV	With			Without			Ratio	Adjusted HR	95%CI	95%CI	P
Variables	Event	PYs	Rate (per 10^5^ PYs)	Event	PYs	Rate (per 10^5^ PYs)					
**Total**	647	228,486.24	283.17	978	1,983,509.96	49.31	5.743	5.103	4.585	5.678	<0.001
**Gender**											
Male	395	144,922.93	272.56	640	1,265,581.36	50.57	5.390	4.825	4.216	5.521	<0.001
Female	252	83,563.30	301.57	338	717,928.60	47.08	6.405	5.582	4.686	6.651	<0.001
**Age group (years)**											
20–29	33	6,456.16	511.14	14	28,548.24	49.04	10.423	8.967	4.716	17.050	<0.001
30–39	71	20,412.07	347.83	53	127,576.93	41.54	8.373	7.940	5.448	11.572	<0.001
40–49	111	34,115.41	325.37	160	223,115.13	71.71	4.537	4.581	3.537	5.933	<0.001
50–59	176	49,194.50	357.76	294	371,528.37	79.13	4.521	4.328	3.542	5.288	<0.001
60–69	125	50,012.10	249.94	256	463,103.63	55.28	4.521	4.061	3.225	5.114	<0.001
≧70	131	68,296.00	191.81	201	769,637.65	26.12	7.345	6.426	5.070	8.145	<0.001
**Diabetes mellitus (DM)**											
Without	529	183,848.75	287.74	740	1,556,145.80	47.55	6.051	5.421	4.809	6.111	<0.001
With	118	44,637.48	264.35	238	427,364.15	55.69	4.747	3.958	3.117	5.026	<0.001
**Hypertension**											
Without	533	187,115.65	284.85	745	1,466,869.58	50.79	5.609	5.016	4.456	5.646	<0.001
With	114	41,371.18	275.55	233	516,640.38	45.10	6.110	5.446	4.253	6.976	<0.001
**Depression**											
Without	639	225,164.07	283.79	974	1,960,769.42	49.67	5.713	5.065	4.550	5.638	<0.001
With	8	3,322.17	240.81	4	22,740.53	17.59	13.690	15.114	4.027	56.720	<0.001
**Stroke**											
Without	628	217,345.56	288.94	935	1,793,066.24	52.15	5.541	5.019	4.503	5.594	<0.001
With	19	11,140.67	170.55	43	190,443.72	22.58	7.553	7.721	4.317	13.608	<0.001
**Chronic Kidney Disease (CKD)**											
Without	634	220,596.40	287.40	945	1,917,281.65	49.29	5.831	5.208	4.673	5.804	<0.001
With	13	7,889.64	164.77	33	66,228.30	49.83	3.307	2.356	1.188	4.672	0.014
**Osteoporosis**											
Without	645	227,630.31	283.35	974	1,970,764.78	49.42	5.733	5.094	4.578	5.669	<0.001
With	2	855.92	233.67	4	12,745.17	31.38	7.445	8.052	1.333	48.659	0.023
**Nephritis, nephrotic syndrome, and nephrosis**											
Without	609	210,808.81	288.89	929	1,839,943.17	50.49	5.722	5.144	4.608	5.742	<0.001
With	38	17,677.42	214.96	49	143,566.78	34.13	6.298	4.540	2.873	7.174	<0.001
**Hyperlipidaemia**											
Without	609	224,485.09	271.29	929	1,905,167.63	48.76	5.563	4.944	4.436	5.510	<0.001
With	38	4,001.15	949.73	49	78,342.32	62.55	15.184	12.440	6.860	22.558	<0.001
**Systemic lupus erythematosus (SLE)**											
Without	644	227,931.26	282.54	975	1,980,062.17	49.24	5.738	5.109	4.591	5.686	<0.001
With	3	554.97	540.57	3	3,447.79	87.01	6.213	4.032	0.701	23.198	0.118

PYs, person-years; adjusted HR, adjusted hazard ratio: adjusted for the variables listed in the Cox regression table; CI, confidence interval; HBV, hepatitis B virus; HCV, hepatitis C virus.

### Comparison of HRs among the HBV/HCV, HBV, and HCV groups for the risk of SSNHL

Cox proportional hazards regressions analysis revealed an increased risk of SSNHL, with an incidence rate ratio (IRR) of 5.743 (95% CI, 4.585–5.678) in the HBV/HCV group, after adjustment for sex, age, and comorbidities. In addition, the individual IRRs for SSNHL in the subgroups of HBV (3.24) and HCV (2.503) were identified. All adjusted HRs for the risk of SSNHL were similar among the HBV/HCV (5.103; 95% CI, 4.585–5.678), HBV (5.110; 95% CI, 4.495–5.809), and HCV (5.094; 95% CI, 4.437–5.848) groups compared with the control group (P < 0.001; Table 3).

**Table 3:** Factors for sudden sensorineural hearing loss in the HBV/HCV group and the HBV and HCV subgroups at the end of follow-up, assessed through Cox regression analysis.

HBV / HCV	With			Without			Ratio	Adjusted HR	95%CI	95%CI	P
Subgroup	Event	PYs	Rate (per 10^5^ PYs)	Event	PYs	Rate (per 10^5^ PYs)					
**Total**	647	228,486.24	283.17	978	1,983,509.96	49.31	5.743	5.103	4.585	5.678	<0.001
**HBV**	365	228,486.24	159.75	978	1,983,509.96	49.31	3.240	5.110	4.495	5.809	<0.001
**HCV**	282	228,486.24	123.42	978	1,983,509.96	49.31	2.503	5.094	4.437	5.848	<0.001

PYs, person-years; adjusted HR, adjusted hazard ratio: adjusted for the variables listed in the Cox regression table; CI, confidence interval; HBV, hepatitis B virus; HCV, hepatitis C virus.

## Discussion

To the best of our knowledge, this is the first large-scale retrospective matched-cohort study to explore the association between SSNHL and HBV/HCV infection. The main finding of this study was that patients who were diagnosed as having HBV/HCV infections between January 1, 2000, and December 31, 2010, had a significantly higher incidence of SSNHL, with an IRR of 5.743 (P < 0.001), than did the general population without HBV/HCV infections.

The prevalence of HBV/HCV infections varies from <0.5% in Western countries to 8%–25% in endemic countries in East Asia [19]. In our study, the prevalence of HBV/HCV infections was 17.4%. Compared with females, males were predominantly infected with HBV/HCV (110,150 males vs. 60,792 females). The age at diagnosis of HBV/HCV infection was usually >40 years, which was compatible with a previous report describing a mean age at diagnosis of 42 years; 60% of these were male patients [20]. In addition, our study demonstrated that the development of SSNHL was most common in patients aged ≥40 years, and that most patients with development of SSNHL were men (395 males vs. 252 females).

At the end of follow-up, all comorbidities were significantly differentially distributed between the HBV/HCV and control groups (Table 1). In the HVB/HCV group, a lower rate of the participants with individual comorbidity was observed for most comorbidities, whereas for depression and SLE, a higher rate of the participants was observed compared with the control group. A higher frequency of some degree of depression was reported in both hepatitis B and C patients [21]. However, our finding of a higher percentage of SLE in patients with HBV/HCV infection was inconsistent with previous results [22]. Additional in-depth investigations are warranted to explore the codependence among these comorbidities and HBV/HCV infections through multivariate analyses.

Our data revealed that the incidence of SSNHL was approximately two-fold higher in the HBV/HCV group than in the control group, and that this difference was significant. The general estimated incidence of SSNHL varies from 5 to 20 cases per 100,000 per year [1]. In our study, the annual incidence of SSNHL was approximately 38 cases per 100,000 in the HBV/HCV group compared with 19 cases per 100,000 in the control group. The incidence noted in our study was relatively high because the use of a nationwide and large-scale survey may have increased the number of patients treated for SSNHL in outpatient and inpatient departments. In a previous report, the annual SSNHL incidence in Taiwan ranged between 6.49 and 10.21 per 100,000; this number included only the SSNHL patients with hospital admission [23].

Despite the association between HBV/HCV infection and several risk factors for SSNHL, the risk of SSNHL remained significantly higher in the HBV/HCV infection cohort, after adjustment for sex, age group, and comorbidities. The association between HBV/HCV infections and SSNHL may be because of shared risk factors. However, we can confidently claim that the increased risk of SSNHL in these patients was likely the effect of HBV/HCV infection, because the possible confounding factors for SSNHL were already substantially adjusted for in this study. Even in the subgroups of HBV or HCV infection alone, a significantly increased risk of SSNHL (with a higher adjusted HR) was observed compared with the control group. These data indicate that hepatitis virus infection has a very strong impact on the risk of SSNHL.

The underlying mechanisms linking HBV/HCV infections with SSNHL development remain unclear. We hypothesized that, in patients with HBV/BCV infections, SSNHL could occur due to an acute exacerbation of viral hepatitis and subsequent SNHL [8] or a chronic viral reaction causing chronic hearing loss [24, 25]. Viruses could gain access to the inner ear via the hematogenous route and induce severe pathophysiologic changes or an immune-mediated reaction [8]. HBV/HCV infections can stimulate the production of inflammatory cytokines such as tumor necrosis factor-alpha, interleukin-1, and interleukin-6, which are injurious to the cochlear hair cells [26]. In addition, hepatitis virus infection has a well-documented association with polyarteritis nodosa, which is a life-threatening necrotizing vasculitis that may result in hearing loss [24, 27].

Our results demonstrate that a greatly increased risk of SSNHL was observed in patients with HBV/HCV infections, according to the data of a large population (170,942 patients with HBV/HCV and 512,826 controls) selected from a retrospective matched-cohort comprising 1,000,000 people covered by the National Health Insurance program; the large sample size benefitted the statistical analysis. This large data resource enables us to investigate the risk factors for SSNHL in Taiwan, with an acceptable selection bias and an enhanced statistical precision.

Our research has several shortcomings. First, several potentially confounding risk factors, such as alcohol consumption, smoking, ototoxic drug effects, and noise exposure for SSNHL, were unavailable in the data resource, which may have led to a certain bias [28]. Second, the database did not provide audiometric results regarding the degree of hearing impairment or routine blood and biochemistry tests data. Third, a population-based study cannot clarify the real mechanism underlying the association between HBV/HCV infection and SSNHL because extracting cochlear tissue pathogens or detecting cochlear injury through imaging is very difficult [7, 8]. In addition, patients who developed SSNHL due an ototoxic effect after antiviral drug administration for HVB/HCV infection (which has been reported in some studies) could not be excluded [10, 11]. However, a case–control study revealed pegylated interferon plus ribavirin therapy does not have any impact on the hearing thresholds of patients with HCV [29]. Finally, we could not exclude the possibility of virus-unrelated hepatitis, such as alcohol-related hepatitis, being correlated with the risk of SSNHL. Additional large-scale studies need to be performed to clarify the discrepancy between virus-induced SSNHL and hepatitis-induced SSNHL. Despite these limitations, this study contributes to the awareness of the increased risk of SSNHL in HBV/HCV-infected populations.

## Conclusions

In this study, HBV/HCV infections present a clearly elevated risk for SSNHL. Regular audiometric tests are recommended for patients with HBV/HCV infection to assess their hearing ability and enable the earlier detection of SSNHL. We also suggest that HBV or HCV carriers presenting with the sudden onset of hearing loss should be examined for the possibility of acute exacerbation of chronic HBV/HCV infection.
